# 1-Methyl-3-(naphthalen-1-yl)-3,3a,4,9b-tetra­hydro-1*H*-chromeno[4,3-*c*]isoxazole-3a-carbonitrile

**DOI:** 10.1107/S1600536811009731

**Published:** 2011-03-23

**Authors:** Rajeswari Gangadharan, K. SethuSankar, Gandhi Murugan, Manickam Bakthadoss

**Affiliations:** a Department of Physics, Ethiraj College for Women (Autonomous), Chennai 600 008, India; b Department of Physics, R.K.M. Vivekananda College (Autonomous), Chennai 600 004, India; c Department of Organic Chemistry, University of Madras, Maraimalai Campus, Chennai 600 025, India

## Abstract

In the title compound, C_22_H_18_N_2_O_2_, the pyran ring of the chromene unit is fused with an isoxazole ring, which adopts an N-envelope conformation with the N atom lying 1.3291 (14) Å from the mean plane of the remaining ring atoms [maximum deviation = 0.341 (2) Å]. The dihedral angle between the isoxazole and chromene units is 43.74 (8)° and that between the iosxazole ring and the naphthalene ring system is 58.82 (8)°. In the crystal, the molecules are linked by weak C—H⋯π inter­actions.

## Related literature

For uses of isoxazole derivatives, see: Baraldi *et al.* (1987 ▶); Eddington *et al.* (2002 ▶); Caine (1993 ▶). For related structures, see: Swaminathan *et al.* (2011*a* ▶,*b* ▶). For puckering parameters, see: Cremer & Pople (1975 ▶). For the synthesis of isoxazolidines, see: Bakthadoss & Murugan (2010 ▶).
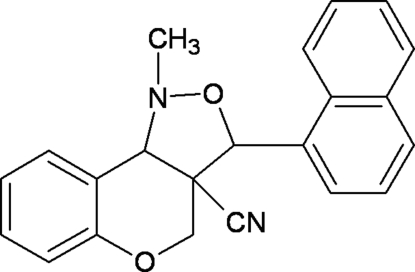


## Experimental

### 

#### Crystal data

C_22_H_18_N_2_O_2_
*M* *_r_* = 342.38
Monoclinic, 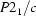

*a* = 10.622 (6) Å
*b* = 12.969 (7) Å
*c* = 12.423 (7) Å
β = 93.64 (3)°
*V* = 1707.9 (16) Å^3^
*Z* = 4
Mo *K*α radiation
μ = 0.09 mm^−1^
*T* = 293 K
0.30 × 0.25 × 0.20 mm

#### Data collection

Bruker APEXII CCD area-detector diffractometer
19983 measured reflections
4684 independent reflections
2767 reflections with *I* > 2σ(*I*)
*R*_int_ = 0.054

#### Refinement

*R*[*F* ^2^ > 2σ(*F* ^2^)] = 0.054
*wR*(*F* ^2^) = 0.176
*S* = 1.03
4684 reflections
236 parameters
H-atom parameters constrained
Δρ_max_ = 0.26 e Å^−3^
Δρ_min_ = −0.31 e Å^−3^

### 

Data collection: *APEX2* (Bruker, 2004 ▶); cell refinement: *SAINT* (Bruker, 2004 ▶); data reduction: *SAINT*; program(s) used to solve structure: *SHELXS97* (Sheldrick, 2008 ▶); program(s) used to refine structure: *SHELXL97* (Sheldrick, 2008 ▶); molecular graphics: *ORTEP-3* (Farrugia, 1997 ▶); software used to prepare material for publication: *SHELXL97* and *PLATON* (Spek, 2009 ▶).

## Supplementary Material

Crystal structure: contains datablocks global, I. DOI: 10.1107/S1600536811009731/pv2398sup1.cif

Structure factors: contains datablocks I. DOI: 10.1107/S1600536811009731/pv2398Isup2.hkl

Additional supplementary materials:  crystallographic information; 3D view; checkCIF report

## Figures and Tables

**Table 1:** Hydrogen-bond geometry (Å, °) *Cg*4 is the centroid of the C12–C17 ring.

*D*—H⋯*A*	*D*—H	H⋯*A*	*D*⋯*A*	*D*—H⋯*A*
C11—H11*C*⋯*Cg*4^i^	0.96	2.84	3.477 (2)	125

Symmetry code: (i) 
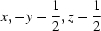
.

**Fig. 1.**
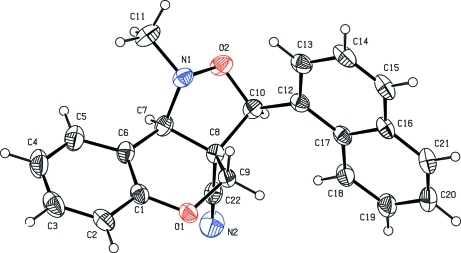
The molecular structure of title compound showing 30% probability displacement ellipsoids.

## supplementary crystallographic information

### Comment

Isoxazole and its derivates are key intermediates for the preparation of products which mimic natural compounds (Baraldi *et al.*, 1987). They have been shown to possess anticonvulsant activity (Eddington  *et al.*, 2002). Chromenopyrrole compounds are used in the treatment of impulsive disorders (Caine, 1993).

In the title compound (Fig. 1), the isoxazole rings adopts an N1-envelope conformation; N1 lies 1.3291 (14) Å from the mean plane formed by the rest of the ring atoms. The dihedral angle between the isoxazole and chromeno moiety is 43.74 (8)°. The dihedral angle between the isoxazole and naphthalene ring system is 58.82 (8)°, indicating a bisectional orientation. The pyran ring (O1/C1/C6-C9) adopts a half-chair conformation with puckering amplitudes (Cremer & Pople, 1975): *Q* = 0.480 (2) Å, θ = 129.2 (2)° and φ = 101.2 (2)°. The title compound exhibits structural similarities with other reported structures (Swaminathan *et al.*, 2011**a*,b*). The molecular structure is stabilized by C–H···π interactions (C11–H11C···Cg4 where Cg4 is the centroid of the six membered ring defined by the atoms C12–C17) (Table 1).

### Experimental

A mixture of (*E*)-2-((2-formylnaphthalen-3-yloxy) -3-phenylacrylonitrile (1 mmol), *N*-methylhydroxyamine hydrochloride (1.1 mmol), pyridine (0.24 mL, 3 mmol) and ethanol (5 mL) was placed in a round bottom flask and refluxed for 6 h. After completion of the reaction as indicated by TLC the reaction mixture was concentrated under reduced pressure. The crude product was diluted with water (10 ml), dilute HCl (5 mL) and extracted with ethylacetate (20 ml). The organic layer was washed with brine solution (10 ml) and concentrated. The crude product was purified by column chromatography to provide the pure title compound as colourless solid. Crystals of the title compound were grown from its solution in methanol by slow evaporation at room temperature.

### Refinement

Hydrogen atoms were placed in calculated positions with C—H = 0.93, 0.96, 0.97 and 0.98 Å for aryl, methyl, methylene and methyne type H-atoms, respectively, and refined in riding model with fixed isotropic displacement parameters: *U*_iso_(H) = 1.5 *U*_eq_(methyl-C) and *U*_iso_(H) = 1.2 *U*_eq_(the rest of the C atoms).

### Crystal data

**Table:**

C_22_H_18_N_2_O_2_	*F*(000) = 720
*M**_r_* = 342.38	*D*_x_ = 1.332 Mg m^−^^3^
Monoclinic, *P*2_1_/*c*	Mo *K*α radiation, λ = 0.71073 Å
Hall symbol: -P 2ybc	Cell parameters from 4684 reflections
*a* = 10.622 (6) Å	θ = 1.0–25.0°
*b* = 12.969 (7) Å	µ = 0.09 mm^−^^1^
*c* = 12.423 (7) Å	*T* = 293 K
β = 93.64 (3)°	Block, colourless
*V* = 1707.9 (16) Å^3^	0.30 × 0.25 × 0.20 mm
*Z* = 4	

### Data collection

**Table:**

Bruker APEXII CCD area-detector diffractometer	2767 reflections with *I* > 2σ(*I*)
Radiation source: fine-focus sealed tube	*R*_int_ = 0.054
graphite	θ_max_ = 29.6°, θ_min_ = 2.3°
ω and φ scans	*h* = −13→14
19983 measured reflections	*k* = −17→13
4684 independent reflections	*l* = −17→17

### Refinement

**Table:**

Refinement on *F*^2^	Primary atom site location: structure-invariant direct methods
Least-squares matrix: full	Secondary atom site location: difference Fourier map
*R*[*F*^2^ > 2σ(*F*^2^)] = 0.054	Hydrogen site location: inferred from neighbouring sites
*wR*(*F*^2^) = 0.176	H-atom parameters constrained
*S* = 1.03	*w* = 1/[σ^2^(*F*_o_^2^) + (0.0917*P*)^2^] where *P* = (*F*_o_^2^ + 2*F*_c_^2^)/3
4684 reflections	(Δ/σ)_max_ < 0.001
236 parameters	Δρ_max_ = 0.26 e Å^−^^3^
0 restraints	Δρ_min_ = −0.31 e Å^−^^3^

### Special details

**Table:**

Geometry. All e.s.d.'s (except the e.s.d. in the dihedral angle between two l.s. planes) are estimated using the full covariance matrix. The cell e.s.d.'s are taken into account individually in the estimation of e.s.d.'s in distances, angles and torsion angles; correlations between e.s.d.'s in cell parameters are only used when they are defined by crystal symmetry. An approximate (isotropic) treatment of cell e.s.d.'s is used for estimating e.s.d.'s involving l.s. planes.
Refinement. Refinement of *F*^2^ against ALL reflections. The weighted *R*-factor *wR* and goodness of fit *S* are based on *F*^2^, conventional *R*-factors *R* are based on *F*, with *F* set to zero for negative *F*^2^. The threshold expression of *F*^2^ > σ(*F*^2^) is used only for calculating *R*-factors(gt) *etc*. and is not relevant to the choice of reflections for refinement. *R*-factors based on *F*^2^ are statistically about twice as large as those based on *F*, and *R*- factors based on ALL data will be even larger.

### Fractional atomic coordinates and isotropic or equivalent isotropic displacement parameters (Å^2^)

**Table:**

	*x*	*y*	*z*	*U*_iso_*/*U*_eq_	
C1	0.14265 (14)	0.10109 (13)	0.82608 (15)	0.0435 (4)	
C2	0.09651 (16)	0.01485 (15)	0.77009 (18)	0.0565 (5)	
H2	0.0678	0.0207	0.6981	0.068*	
C3	0.09339 (19)	−0.07866 (16)	0.8210 (2)	0.0711 (7)	
H3	0.0634	−0.1364	0.7831	0.085*	
C4	0.1342 (2)	−0.08753 (16)	0.9273 (2)	0.0764 (7)	
H4	0.1317	−0.1510	0.9619	0.092*	
C5	0.17889 (19)	−0.00189 (15)	0.98249 (19)	0.0634 (5)	
H5	0.2053	−0.0082	1.0550	0.076*	
C6	0.18571 (15)	0.09400 (13)	0.93287 (14)	0.0445 (4)	
C7	0.23100 (15)	0.18759 (13)	0.99341 (13)	0.0423 (4)	
H7	0.1947	0.1897	1.0640	0.051*	
C8	0.20434 (14)	0.28953 (12)	0.93319 (12)	0.0378 (4)	
C9	0.22192 (14)	0.27038 (13)	0.81394 (12)	0.0392 (4)	
H9A	0.2069	0.3340	0.7741	0.047*	
H9B	0.3082	0.2492	0.8051	0.047*	
C10	0.30763 (15)	0.36307 (13)	0.98762 (13)	0.0429 (4)	
H10	0.2653	0.4150	1.0298	0.051*	
C11	0.4298 (2)	0.12639 (17)	1.08402 (16)	0.0642 (5)	
H11A	0.5172	0.1446	1.0957	0.096*	
H11B	0.4231	0.0572	1.0567	0.096*	
H11C	0.3896	0.1307	1.1509	0.096*	
C12	0.39286 (14)	0.41856 (12)	0.91194 (13)	0.0401 (4)	
C13	0.51461 (16)	0.38596 (14)	0.90491 (16)	0.0503 (4)	
H13	0.5434	0.3293	0.9452	0.060*	
C14	0.59706 (16)	0.43657 (15)	0.83792 (19)	0.0583 (5)	
H14	0.6797	0.4135	0.8349	0.070*	
C15	0.55729 (17)	0.51739 (15)	0.77876 (18)	0.0571 (5)	
H15	0.6122	0.5493	0.7338	0.069*	
C16	0.43293 (16)	0.55539 (13)	0.78330 (15)	0.0478 (4)	
C17	0.34944 (14)	0.50587 (12)	0.85222 (14)	0.0407 (4)	
C18	0.22757 (16)	0.54867 (14)	0.85861 (17)	0.0520 (5)	
H18	0.1719	0.5188	0.9043	0.062*	
C19	0.19061 (18)	0.63210 (15)	0.7997 (2)	0.0651 (6)	
H19	0.1107	0.6598	0.8066	0.078*	
C20	0.2715 (2)	0.67751 (17)	0.7281 (2)	0.0702 (6)	
H20	0.2438	0.7329	0.6854	0.084*	
C21	0.3899 (2)	0.64047 (16)	0.72136 (19)	0.0632 (5)	
H21	0.4437	0.6718	0.6750	0.076*	
C22	0.07561 (16)	0.32612 (14)	0.94773 (14)	0.0465 (4)	
N1	0.36917 (13)	0.19627 (11)	1.00702 (11)	0.0466 (4)	
N2	−0.02444 (16)	0.35439 (15)	0.95782 (16)	0.0724 (5)	
O1	0.13851 (10)	0.19321 (9)	0.77127 (9)	0.0466 (3)	
O2	0.37728 (12)	0.29730 (9)	1.05968 (9)	0.0549 (4)	

### Atomic displacement parameters (Å^2^)

**Table:**

	*U*^11^	*U*^22^	*U*^33^	*U*^12^	*U*^13^	*U*^23^
C1	0.0362 (8)	0.0388 (10)	0.0562 (10)	−0.0010 (7)	0.0092 (7)	−0.0039 (8)
C2	0.0412 (9)	0.0505 (12)	0.0780 (13)	−0.0054 (8)	0.0061 (8)	−0.0154 (10)
C3	0.0565 (12)	0.0434 (13)	0.114 (2)	−0.0055 (9)	0.0087 (12)	−0.0151 (13)
C4	0.0777 (14)	0.0374 (12)	0.115 (2)	−0.0021 (10)	0.0170 (14)	0.0093 (13)
C5	0.0695 (12)	0.0467 (12)	0.0752 (14)	0.0013 (10)	0.0127 (10)	0.0105 (10)
C6	0.0466 (9)	0.0365 (9)	0.0517 (10)	0.0020 (7)	0.0135 (7)	0.0023 (8)
C7	0.0519 (9)	0.0399 (10)	0.0359 (8)	0.0053 (7)	0.0096 (6)	0.0038 (7)
C8	0.0444 (8)	0.0361 (9)	0.0336 (7)	0.0011 (7)	0.0071 (6)	−0.0016 (6)
C9	0.0431 (8)	0.0393 (9)	0.0354 (8)	−0.0055 (7)	0.0042 (6)	−0.0016 (7)
C10	0.0529 (9)	0.0391 (9)	0.0362 (8)	0.0040 (7)	−0.0003 (7)	−0.0069 (7)
C11	0.0750 (13)	0.0671 (14)	0.0495 (10)	0.0200 (11)	−0.0032 (9)	0.0119 (10)
C12	0.0416 (8)	0.0352 (9)	0.0432 (8)	−0.0020 (7)	−0.0001 (6)	−0.0135 (7)
C13	0.0460 (9)	0.0395 (10)	0.0642 (11)	0.0005 (8)	−0.0052 (8)	−0.0161 (9)
C14	0.0360 (9)	0.0490 (12)	0.0904 (15)	−0.0050 (8)	0.0080 (9)	−0.0267 (11)
C15	0.0482 (10)	0.0473 (11)	0.0779 (14)	−0.0165 (9)	0.0190 (9)	−0.0205 (10)
C16	0.0469 (9)	0.0389 (10)	0.0581 (10)	−0.0141 (8)	0.0074 (7)	−0.0119 (8)
C17	0.0406 (8)	0.0335 (9)	0.0481 (9)	−0.0050 (7)	0.0029 (7)	−0.0130 (7)
C18	0.0459 (9)	0.0388 (10)	0.0722 (12)	−0.0047 (8)	0.0100 (8)	0.0003 (9)
C19	0.0521 (11)	0.0408 (11)	0.1023 (18)	0.0000 (9)	0.0031 (10)	0.0085 (11)
C20	0.0728 (13)	0.0424 (12)	0.0946 (17)	−0.0101 (10)	−0.0002 (11)	0.0163 (12)
C21	0.0678 (12)	0.0468 (12)	0.0761 (14)	−0.0185 (10)	0.0128 (10)	0.0061 (10)
C22	0.0514 (10)	0.0411 (10)	0.0484 (9)	0.0018 (8)	0.0144 (7)	0.0042 (8)
N1	0.0569 (8)	0.0431 (8)	0.0391 (7)	0.0073 (7)	−0.0033 (6)	−0.0012 (6)
N2	0.0580 (10)	0.0734 (13)	0.0884 (14)	0.0138 (9)	0.0257 (9)	0.0095 (10)
O1	0.0499 (7)	0.0446 (7)	0.0446 (6)	−0.0111 (5)	−0.0027 (5)	−0.0023 (5)
O2	0.0737 (8)	0.0497 (8)	0.0392 (6)	0.0062 (6)	−0.0134 (6)	−0.0071 (6)

### Geometric parameters (Å, °)

**Table:**

C1—O1	1.374 (2)	C11—N1	1.440 (2)
C1—C6	1.379 (3)	C11—H11A	0.9600
C1—C2	1.390 (3)	C11—H11B	0.9600
C2—C3	1.369 (3)	C11—H11C	0.9600
C2—H2	0.9300	C12—C13	1.369 (2)
C3—C4	1.369 (4)	C12—C17	1.415 (2)
C3—H3	0.9300	C13—C14	1.408 (3)
C4—C5	1.374 (3)	C13—H13	0.9300
C4—H4	0.9300	C14—C15	1.333 (3)
C5—C6	1.392 (3)	C14—H14	0.9300
C5—H5	0.9300	C15—C16	1.414 (3)
C6—C7	1.491 (2)	C15—H15	0.9300
C7—N1	1.471 (2)	C16—C21	1.405 (3)
C7—C8	1.537 (2)	C16—C17	1.424 (2)
C7—H7	0.9800	C17—C18	1.415 (2)
C8—C22	1.469 (2)	C18—C19	1.351 (3)
C8—C9	1.525 (2)	C18—H18	0.9300
C8—C10	1.574 (2)	C19—C20	1.405 (3)
C9—O1	1.4171 (19)	C19—H19	0.9300
C9—H9A	0.9700	C20—C21	1.354 (3)
C9—H9B	0.9700	C20—H20	0.9300
C10—O2	1.411 (2)	C21—H21	0.9300
C10—C12	1.526 (2)	C22—N2	1.139 (2)
C10—H10	0.9800	N1—O2	1.4647 (19)
			
O1—C1—C6	122.21 (16)	N1—C11—H11A	109.5
O1—C1—C2	116.89 (17)	N1—C11—H11B	109.5
C6—C1—C2	120.85 (18)	H11A—C11—H11B	109.5
C3—C2—C1	120.0 (2)	N1—C11—H11C	109.5
C3—C2—H2	120.0	H11A—C11—H11C	109.5
C1—C2—H2	120.0	H11B—C11—H11C	109.5
C2—C3—C4	120.3 (2)	C13—C12—C17	119.45 (16)
C2—C3—H3	119.8	C13—C12—C10	119.56 (16)
C4—C3—H3	119.8	C17—C12—C10	120.94 (14)
C3—C4—C5	119.5 (2)	C12—C13—C14	121.28 (18)
C3—C4—H4	120.3	C12—C13—H13	119.4
C5—C4—H4	120.3	C14—C13—H13	119.4
C4—C5—C6	121.8 (2)	C15—C14—C13	120.33 (17)
C4—C5—H5	119.1	C15—C14—H14	119.8
C6—C5—H5	119.1	C13—C14—H14	119.8
C1—C6—C5	117.54 (18)	C14—C15—C16	121.06 (18)
C1—C6—C7	120.50 (15)	C14—C15—H15	119.5
C5—C6—C7	121.87 (17)	C16—C15—H15	119.5
N1—C7—C6	114.07 (13)	C21—C16—C15	121.58 (17)
N1—C7—C8	98.15 (12)	C21—C16—C17	119.37 (16)
C6—C7—C8	114.28 (14)	C15—C16—C17	119.05 (18)
N1—C7—H7	109.9	C12—C17—C18	123.59 (15)
C6—C7—H7	109.9	C12—C17—C16	118.78 (15)
C8—C7—H7	109.9	C18—C17—C16	117.61 (16)
C22—C8—C9	110.13 (13)	C19—C18—C17	121.28 (17)
C22—C8—C7	111.24 (13)	C19—C18—H18	119.4
C9—C8—C7	107.62 (13)	C17—C18—H18	119.4
C22—C8—C10	112.35 (14)	C18—C19—C20	120.70 (19)
C9—C8—C10	113.02 (13)	C18—C19—H19	119.7
C7—C8—C10	102.15 (13)	C20—C19—H19	119.7
O1—C9—C8	111.48 (12)	C21—C20—C19	119.9 (2)
O1—C9—H9A	109.3	C21—C20—H20	120.1
C8—C9—H9A	109.3	C19—C20—H20	120.1
O1—C9—H9B	109.3	C20—C21—C16	121.04 (18)
C8—C9—H9B	109.3	C20—C21—H21	119.5
H9A—C9—H9B	108.0	C16—C21—H21	119.5
O2—C10—C12	111.60 (14)	N2—C22—C8	179.3 (2)
O2—C10—C8	103.26 (14)	C11—N1—O2	104.78 (14)
C12—C10—C8	116.47 (13)	C11—N1—C7	115.42 (15)
O2—C10—H10	108.4	O2—N1—C7	98.62 (12)
C12—C10—H10	108.4	C1—O1—C9	115.41 (13)
C8—C10—H10	108.4	C10—O2—N1	104.11 (11)
			
O1—C1—C2—C3	−177.56 (15)	C8—C10—C12—C17	77.62 (19)
C6—C1—C2—C3	0.0 (3)	C17—C12—C13—C14	−1.1 (2)
C1—C2—C3—C4	0.8 (3)	C10—C12—C13—C14	−178.53 (15)
C2—C3—C4—C5	−0.3 (3)	C12—C13—C14—C15	−0.6 (3)
C3—C4—C5—C6	−0.9 (3)	C13—C14—C15—C16	1.2 (3)
O1—C1—C6—C5	176.25 (14)	C14—C15—C16—C21	−179.99 (18)
C2—C1—C6—C5	−1.2 (2)	C14—C15—C16—C17	−0.2 (3)
O1—C1—C6—C7	−0.5 (2)	C13—C12—C17—C18	−176.50 (16)
C2—C1—C6—C7	−177.92 (15)	C10—C12—C17—C18	0.9 (2)
C4—C5—C6—C1	1.7 (3)	C13—C12—C17—C16	2.1 (2)
C4—C5—C6—C7	178.34 (18)	C10—C12—C17—C16	179.54 (14)
C1—C6—C7—N1	−102.19 (17)	C21—C16—C17—C12	178.31 (16)
C5—C6—C7—N1	81.2 (2)	C15—C16—C17—C12	−1.5 (2)
C1—C6—C7—C8	9.6 (2)	C21—C16—C17—C18	−3.0 (2)
C5—C6—C7—C8	−166.94 (15)	C15—C16—C17—C18	177.18 (16)
N1—C7—C8—C22	−155.46 (13)	C12—C17—C18—C19	−179.89 (18)
C6—C7—C8—C22	83.44 (17)	C16—C17—C18—C19	1.5 (3)
N1—C7—C8—C9	83.82 (14)	C17—C18—C19—C20	1.5 (3)
C6—C7—C8—C9	−37.28 (17)	C18—C19—C20—C21	−3.0 (4)
N1—C7—C8—C10	−35.39 (14)	C19—C20—C21—C16	1.4 (3)
C6—C7—C8—C10	−156.49 (13)	C15—C16—C21—C20	−178.6 (2)
C22—C8—C9—O1	−61.53 (18)	C17—C16—C21—C20	1.6 (3)
C7—C8—C9—O1	59.89 (17)	C6—C7—N1—C11	−73.38 (19)
C10—C8—C9—O1	171.90 (13)	C8—C7—N1—C11	165.36 (14)
C22—C8—C10—O2	122.56 (14)	C6—C7—N1—O2	175.66 (12)
C9—C8—C10—O2	−112.06 (14)	C8—C7—N1—O2	54.40 (13)
C7—C8—C10—O2	3.27 (15)	C6—C1—O1—C9	23.1 (2)
C22—C8—C10—C12	−114.77 (15)	C2—C1—O1—C9	−159.34 (14)
C9—C8—C10—C12	10.62 (19)	C8—C9—O1—C1	−53.94 (18)
C7—C8—C10—C12	125.94 (14)	C12—C10—O2—N1	−94.92 (14)
O2—C10—C12—C13	13.2 (2)	C8—C10—O2—N1	30.93 (15)
C8—C10—C12—C13	−104.98 (17)	C11—N1—O2—C10	−174.85 (14)
O2—C10—C12—C17	−164.17 (13)	C7—N1—O2—C10	−55.57 (15)

### Hydrogen-bond geometry (Å, °)

**Table:**

Cg4 is the centroid of the C12–C17 ring.

**Table:**

*D*—H···*A*	*D*—H	H···*A*	*D*···*A*	*D*—H···*A*
C11—H11C···Cg4^i^	0.96	2.84	3.477 (2)	125

Symmetry codes: (i) *x*, −*y*−1/2, *z*−1/2.
